# Frequency and genome load of Epstein-Barr virus in 509 breast cancers from different geographical areas[Fn tlnote1]

**DOI:** 10.1054/bjoc.2000.1672

**Published:** 2001-03

**Authors:** F Fina, S Romain, L'H Ouafik, J Palmari, F Ben Ayed, S Benharkat, P Bonnier, F Spyratos, J A Foekens, C Rose, M Buisson, H Gérard, M O Reymond, J M Seigneurin, P M Martin

**Affiliations:** Assistance Publique-Hôpitaux de Marseille, Laboratoire de Transfert d'Oncologie Biologique, Faculté de Médecine Nord, Boulevard Pierre Dramard, 13916 Marseille Cedex 20, France; Institut Salah Azaïz, Service d'Hémato-Oncologie, Boulevard du 9 Avril 1938, BP 173, 1006 Tunis, Tunisia; Centre Hospitalo-Universitaire Ibn Rochd, Laboratoire Central, 23000 Annaba, Algeria; Assistance Publique-Hôpitaux de Marseille, Service de Gynécologie et Obstétrique A, Hôpital de la Conception, 147 Boulevard Baille, 13385 Marseille Cedex 5, France; Centre René Huguenin, Département de Biologie, 35 rue Dailly, 92210 St Cloud, France; Josephine Nefkens Institute, Dr. Molewaterplein 50, room Be426, 3015 GE Rotterdam, The Netherlands; University Hospital, Department of Oncology R, DK-5000 Odense C, Denmark; Faculté de Médecine de Grenoble, Laboratoire de Virologie Médicale Moléculaire, Domaine de la Merci, 38706 La Tronche, France

**Keywords:** breast cancer, Epstein–Barr virus, polymerase chain reaction, real-time quantitative polymerase chain reaction, genotyping, in situ hybridization, laser capture microdissection

## Abstract

Since the few data exploring a possible association between Epstein–Barr virus (EBV) and breast cancer are conflicting, we investigated this association together with the influences of geographical areas. 509 breast cancers were sampled from areas with varying risks of nasopharynx carcinoma (NPC) such as North Africa (Algeria and Tunisia, high-risk area); southern France (Marseille, intermediate-risk area); and northern Europe (northern France, the Netherlands and Denmark; low-risk areas). Polymerase chain reaction (PCR) of a subregion of EBV BamHIC encoding the EBERs demonstrated that 31.8% of the tumours contained the viral genome. No significant differences were observed among the geographical areas. However, positive samples showed higher loads of the EBV genome in the NPC high- and intermediate-risk areas than in the low-risk areas. EBV type 1 was the dominant strain. In situ hybridization studies using a^35^S-labelled riboprobe for EBER1 and a laser capture microdissection, combined with quantitative PCR, showed that EBV localization was restricted to some tumour epithelial cell clusters. EBV could not be detected in the stroma. Considering the whole population covered, the presence of the EBV genome was not correlated with age, menopausal status, tumour, size, nodal status or histological grade. © 2001 Cancer Research Campaign http://www. bjcancer.com


*British Journal of Cancer* (2001) **84**(6), 783–790
© 2001 Cancer Research Campaign
doi: 10.1054/ bjoc.2000.1672, available online at http://www.idealibrary.com on IDEAL®

http://www.bjcancer.com

### Frequency and genome load of Epstein–Barr virus in 509 breast cancers from different geographical areas*

**F Fina[1], S Romain[1], L'H Ouafik[1], J Palmari[1], F Ben Ayed[2], S Benharkat[3], P Bonnier[4], F Spyratos[5], JA Foekens[6], C Rose[7], M Buisson[8], H Gérard[1], MO Reymond[1], JM Seigneurin[8] and PM Martin[1]**

[1]Assistance Publique-Hôpitaux de Marseille, Laboratoire de Transfert d'Oncologie Biologique, Faculté de Médecine Nord, Boulevard Pierre Dramard, 13916 Marseille Cedex 20, France; [2]Institut Salah Azaïz, Service d'Hémato-Oncologie, Boulevard du 9 Avril 1938, BP 173, 1006 Tunis, Tunisia; [3]Centre Hospitalo-Universitaire Ibn Rochd, Laboratoire Central, 23000 Annaba, Algeria; [4]Assistance Publique-Hôpitaux de Marseille, Service de Gynécologie et Obstétrique A, Hôpital de la Conception, 147 Boulevard Baille, 13385 Marseille Cedex 5, France; [5]Centre René Huguenin, Département de Biologie, 35 rue Dailly, 92210 St Cloud, France; [6]Josephine Nefkens Institute, Dr. Molewaterplein 50, room Be426, 3015 GE Rotterdam, The Netherlands; [7]University Hospital, Department of Oncology R, DK-5000 Odense C, Denmark; [8]Faculté de Médecine de Grenoble, Laboratoire de Virologie Médicale Moléculaire, Domaine de la Merci, 38706 La Tronche, France

**Summary** Since the few data exploring a possible association between Epstein–Barr virus (EBV) and breast cancer are conflicting, we investigated this association together with the influences of geographical areas. 509 breast cancers were sampled from areas with varying risks of nasopharynx carcinoma (NPC) such as North Africa (Algeria and Tunisia, high-risk area); southern France (Marseille, intermediate-risk area); and northern Europe (northern France, the Netherlands and Denmark; low-risk areas). Polymerase chain reaction (PCR) of a subregion of EBV BamHIC encoding the EBERs demonstrated that 31.8% of the tumours contained the viral genome. No significant differences were observed among the geographical areas. However, positive samples showed higher loads of the EBV genome in the NPC high- and intermediate-risk areas than in the low-risk areas. EBV type 1 was the dominant strain. In situ hybridization studies using a $^{35}$S-labelled riboprobe for EBER1 and a laser capture microdissection, combined with quantitative PCR, showed that EBV localization was restricted to some tumour epithelial cell clusters. EBV could not be detected in the stroma. Considering the whole population covered, the presence of the EBV genome was not correlated with age, menopausal status, tumour, size, nodal status or histological grade. © 2001 Cancer Research Campaign http://www.bjcancer.com

**Keywords**: breast cancer; Epstein–Barr virus; polymerase chain reaction; real-time quantitative polymerase chain reaction; genotyping; in situ hybridization; laser capture microdissection

The links between viruses and cancer have been extensively investigated (zur Hausen, 1991). Epstein–Barr virus (EBV) was the first virus shown to contribute to major cell proliferation disorders in humans. EBV was initially detected in neoplasic cells of endemic Burkitt's lymphoma (BL), undifferentiated nasopharynx carcinoma (NPC), then in Hodgkin's disease. The few data available on EBV in breast tumours are conflicting (Chang et al, 1992; Gaffey et al, 1993; Horiuchi et al, 1994; Labrecque et al, 1995; Lespagnard et al, 1995; Glaser et al, 1998; Bonnet et al, 1999; Brink et al, 2000).

EBV is a γ-herpesvirus. Latent products of its DNA genome include highly abundant small transcripts (EBER 1 and 2) and nuclear proteins (EBNA-1–6). Two types of EBV (EBV1 and EBV2) have been identified based on the divergent sequences in the EBNA genes. In vitro, EBV1 is the more strongly immortalizing of the types (Rickinson et al, 1987).

Serological evidence shows that EBV infects most people early in life and is generally carried in latent form by peripheral B lymphocytes. In contrast, there are large geographical variations in the prevalence of cancers with the strongest link to EBV (Parkin, 1986; De The, 1993). Endemic foci of NPC are found in the Maghreb (Hubert et al, 1993; IARC, 1997), while the risk of NPC is low in northern Europe. The prevalence is intermediate in southern France where migrants of Maghrebian origin and French people born in Maghreb represent more than 20% of the population (Jeannel et al, 1993; Bouchardy et al, 1996).

The aim of this study was to investigate the frequency and genome load of EBV in a large series of breast cancers, alongside possible geographical influences. On behalf of an international study group we collected 509 primary invasive ductal breast cancers from areas with varying risks of NPC. Different techniques were used to assess the presence of EBV in these tumours, including polymerase chain reaction (PCR) to amplify a subregion

*Received 2 August 2000*
*Revised 18 December 2000*
*Accepted 21 December 2000*

*Correspondence to:* S Romain

*Part of this work was presented to the VII Symposium of the International Association for Research on Epstein–Barr Virus and Associated Diseases, Hong-Kong, 13–16 November 1996.

On behalf of: K Rahal, A Gammoudi (Institut Salah Azaïz, Tunis, Tunisia); S Haddad, A Djemaa (Centre Hospitalier Universitaire, Constantine, Algeria); L Piana (Hôpital de la Conception, Marseille, France); JM Brandone, C Bressac (Clinique Bouchard, Marseille, France); C Charpin (Assistance Publique-Hôpitaux de Marseille, Faculté de Médecine Nord, Marseille, France); M Pizzi-Anselme, J Del Grande, J Guidon (Laboratoire d'Anatomie Pathologique et Cytologie, Marseille, France); and the clinicians and pathologists from Centre René Huguenin (St Cloud, France); Dr Daniel den Hoed Cancer Center (Rotterdam, the Netherlands); and the Finsen Institute (Copenhagen, Denmark) who actively participated in the study.

of BamHIC encoding the EBERs, genotyping PCR to determine EBV subtype, in situ hybridization (ISH) of EBV ribonucleic acid and laser capture microdissection (LCM), combined with quantitative PCR (Q-PCR), to localize EBV. The clinical profiles of EBV-negative and EBV-positive tumours were compared.

### MATERIALS AND METHODS

### Patients

This study involved 509 primary invasive ductal breast carcinomas with clinical and pathological characteristics shown in Table 1.

*NPC high-risk areas (Maghrebian countries)*

Patients from Algeria (*n* = 40) were recruited in Annaba and Constantine, and patients from Tunisia (*n* = 58) were treated at the Institut Salah Azaïz. Young patients and patients with inflammatory carcinomas (Sobin and Wittekind, 1997) represented a large proportion of these populations, as previously reported (Tabbane et al, 1977; Tabbane et al, 1985).

*NPC intermediate-risk area (southern France)*

Patients from Marseille, France (*n* = 116) were treated at the Clinique Bouchard and Hôpital de la Conception using similar strategies. A high proportion (19.1%) of patients less than 35 years old was selected.

*NPC low-risk areas (northern Europe)*

Patients from St Cloud, France (*n* = 72) were treated at the Centre René Huguenin. Patients from The Netherlands (*n* = 126) underwent primary surgery in or were referred to the Daniel den Hoed Cancer Center in Rotterdam from 1981 to 1992 for adjuvant radiotherapy; all the patients were node-positive and were included in a

**Table 1** Clinical and pathological characteristics

| Variable | Total *n* = 509 | | Algeria *n* = 40 | | Tunisia *n* = 58 | | Marseille *n* = 116 | | St Cloud *n* = 72 | | Netherlands *n* = 126 | | Denmark *n* = 97 | |
|---|---|---|---|---|---|---|---|---|---|---|---|---|---|---|
| | *n*[1] | % | *n*[1] | % | *n*[1] | % | *n*[1] | % | *n*[1] | % | *n*[1] | % | *n*[1] | % |
| Age (years) | | | | | | | | | | | | | | |
| ≤35 | 63 | 12.4 | 10 | 25.0 | 9 | 15.5 | 22 | 19.1 | 4 | 5.5 | 12 | 9.5 | 6 | 6.2 |
| (35–40) | 79 | 15.6 | 9 | 22.5 | 14 | 24.2 | 21 | 18.3 | 8 | 11.1 | 20 | 15.9 | 7 | 7.2 |
| (40–50) | 143 | 28.1 | 6 | 15.0 | 17 | 29.3 | 23 | 20.0 | 12 | 16.7 | 64 | 50.8 | 21 | 21.7 |
| (50–70) | 182 | 35.8 | 15 | 37.5 | 17 | 29.3 | 28 | 24.3 | 36 | 50.0 | 30 | 23.8 | 56 | 57.7 |
| >70 | 41 | 8.1 | 0 | 0.0 | 1 | 1.7 | 21 | 18.3 | 12 | 16.7 | 0 | 0.0 | 7 | 7.2 |
| Hormonal status | | | | | | | | | | | | | | |
| pre-menopausal | 257 | 64.6 | 25 | 62.5 | 38 | 65.5 | 52 | 51.0 | 36 | 50.0 | 106 | 84.1 | | |
| post-menopausal | 141 | 35.4 | 15 | 37.5 | 20 | 34.5 | 50 | 49.0 | 36 | 50.0 | 20 | 15.9 | | |
| Clinical tumour size | | | | | | | | | | | | | | |
| T0 | 3 | 1.4 | | | 0 | 0.0 | 1 | 1.1 | 2 | 2.9 | | | | |
| T1 | 46 | 21.3 | | | 1 | 1.9 | 19 | 20.7 | 26 | 37.1 | | | | |
| T2 | 107 | 49.6 | | | 18 | 33.3 | 53 | 57.6 | 36 | 51.4 | | | | |
| T3 | 45 | 20.8 | | | 26 | 48.1 | 14 | 15.2 | 5 | 7.2 | | | | |
| T4 | 15 | 6.9 | | | 9 | 16.7 | 5 | 5.4 | 1 | 1.4 | | | | |
| Clinical nodal status | | | | | | | | | | | | | | |
| N0 | 117 | 53.9 | | | 5 | 9.1 | 55 | 60.4 | 57 | 80.3 | | | | |
| N1 | 88 | 40.6 | | | 42 | 76.4 | 33 | 36.3 | 13 | 18.3 | | | | |
| N2 | 10 | 4.6 | | | 6 | 10.9 | 3 | 3.3 | 1 | 1.4 | | | | |
| N3 | 2 | 0.9 | | | 2 | 3.6 | 0 | 0.0 | 0 | 0.0 | | | | |
| Inflammatory status | | | | | | | | | | | | | | |
| not inflammatory | 476 | 95.6 | 30 | 75.0 | 35 | 74.5 | 116 | 100.0 | 72 | 100.0 | 126 | 100.0 | 97 | 100.0 |
| inflammatory | 22 | 4.4 | 10 | 25.0 | 12 | 25.5 | 0 | 0.0 | 0 | 0.0 | 0 | 0.0 | 0 | 0.0 |
| Pathological tumor size (mm) | | | | | | | | | | | | | | |
| <=20 | 128 | 28.9 | | | 2 | 3.7 | 48 | 46.6 | 34 | 47.9 | 39 | 30.9 | 5 | 5.6 |
| (20–50) | 220 | 49.7 | | | 20 | 37.0 | 47 | 45.6 | 31 | 43.7 | 67 | 53.2 | 55 | 61.8 |
| >50 | 95 | 21.4 | | | 32 | 59.3 | 8 | 7.8 | 6 | 8.4 | 20 | 15.9 | 29 | 32.6 |
| Pathological nodal status | | | | | | | | | | | | | | |
| negative | 132 | 27.0 | 15 | 37.5 | 19 | 35.8 | 50 | 47.6 | 37 | 55.2 | 0 | 0.0 | 11 | 11.3 |
| positive | 356 | 73.0 | 25 | 62.5 | 34 | 64.2 | 55 | 52.4 | 30 | 44.8 | 126 | 100.0 | 86 | 88.7 |
| 0 | 117 | 26.1 | | | 19 | 35.8 | 50 | 47.6 | 37 | 55.2 | 0 | 0.0 | 11 | 11.3 |
| 1–3 | 186 | 41.5 | | | 10 | 18.9 | 30 | 28.6 | 22 | 32.9 | 77 | 61.1 | 47 | 48.5 |
| >3 | 145 | 32.4 | | | 24 | 45.3 | 25 | 23.8 | 8 | 11.9 | 49 | 38.9 | 39 | 40.2 |
| Histological grade | | | | | | | | | | | | | | |
| 1 | 53 | 11.8 | 5 | 12.5 | 7 | 13.2 | 18 | 16.5 | 12 | 17.4 | 1 | 1.1 | 10 | 11.2 |
| 2 | 211 | 47.0 | 32 | 80.0 | 28 | 52.8 | 46 | 42.2 | 35 | 50.7 | 15 | 16.9 | 55 | 61.8 |
| 3 | 185 | 41.2 | 3 | 7.5 | 18 | 34.0 | 45 | 41.3 | 22 | 31.9 | 73 | 82.0 | 24 | 27.0 |

*n* = number of patients. [1]Owing to missing values, the number of patients does not always add up to the total number of patients in the analysed population.

 *© 2001 Cancer Research Campaign*

previous study (Romain et al, 1997). Patients from Denmark (*n* = 97) were registered in the high-risk group of the Danish Breast Cancer Cooperative Group program (Andersen et al, 1981).

### Tissue specimens

Breast tumour specimens were obtained at surgery and stored in liquid nitrogen. Samples were all processed in Marseille (laboratoire de Transfert d'Oncologie Biologique, Assistance Publique-Hôpitaux de Marseille). Tumours from other areas were shipped on solid carbon dioxide to this institution. Tumours were prepared for DNA extraction (Sambrook et al, 1989) and cryosections. Fixed paraffin-embedded tissues were obtained in specific cases, according to the results of BamHIC PCR.

### PCR analysis of EBV genome

### *Qualitative BamHIC PCR*

The synthetic primers, sense 5′-AACCTCAGGACCTACGCT-3′ and antisense 5′-TAGCGGACAAGCCGAATAC-3′, described by Labrecque et al (1995), were used for BamHIC PCR. The amplified fragment is 501 base pair (bp). Aliquots of each PCR product were electrophoresed on a 1% agarose gel in Tris/borate buffer containing 0.5 µg $ml^{-1}$ ethidium bromide, photographed, and prepared for Southern blot (Sambrook et al, 1989). The filters were hybridized with a random-primed 200 bp fragment of EBER1 cDNA (kindly provided by BE Griffin and LG Labrecque), washed as previously described (Ouafik et al, 1989), and exposed to film to verify the identity of the bands.

### *Semi-quantitative BamHIC PCR*

To quantify the EBV genome load in the tumours, DNA extracted from the Namalwa cell line, containing two integrated EBV copies per cell, was used. $150 \times 10^3$ Namalwa cells yielded 1 µg of DNA. Serial dilutions of DNA were prepared from 30.0 to 0.1 ng, equivalent to 9000-30 copies of EBV genome, respectively. Data are expressed as the number of BamHIC copies/500 ng DNA.

### *Real-time quantitative BamHIC PCR*

The 5′-exonuclease (TaqMan) assay was used for real-time Q-PCR (Bustin, 2000). Primers for BamHIC were: sense, 5′-AAA-CAG-GAC-AGC-CGT-TGC-C-3′ (6935–6953); antisense, 5′-AAG-CCT-CTC-TTC-TCC-TTC-CCC-3′ (7036–7016), and the probe was FAM-TTT-CGG-ACA-CAC-CGC-CAA-CGC-T-TAMRA (6961–6983). Amplification was performed in a 50 µl reaction volume with a buffer consisting of 10 mmol $l^{-1}$ Tris-HCl (pH 8.3; 25˚C), 50 mmol $l^{-1}$ KC1, 10 mmol $l^{-1}$ ethylenediamine tetraacetate, and 5 mmol $l^{-1}$ $MgCl_2$ in the presence of 200 µmol $l^{-1}$ deoxy (d)-ATP, dCTP and dGTP, 400 µmol $l^{-1}$ dUTP, 200 nmol $l^{-1}$ of each primer, 200 nmol $l^{-1}$ probe, 1 U Amp Erase UNG (Perkin-Elmer Corp, Foster City, USA), and 1.25 U AmpliTaq Gold polymerase (Perkin-Elmer Corp, Foster City, USA). BamHIC levels were normalized to the glyceraldehyde-3-phosphate dehydrogenase (GAPDH). Primers for GAPDH were: sense, 5′-CAA-ATT-CCA-TGG-CAC-CGT-C-3′ (3338–3356); antisense, 5′-GCC-ACA-CCA-TCC-TAG-TTG-C-3′ (3471–3453), and the probe was FAM-CCC-ATC-ACC-ATC-TTC-CAG-TAMRA (3392–3413). PCR reactions were performed on a ABI Prism 7700 sequence detection apparatus (Perkin Elmer Corp, Foster City, USA). The cycling conditions for both BamHIC and GAPDH were as follows: 95˚C for 15 min; 45 cycles of 94˚C for 20 s, 55˚C for 20 s. Data were expressed as the number of BamHIC copies/100 ng GAPDH.

### EBV genotyping

The long divergent region of the EBNA-2 gene, reported for the B95-8 (EBV1) and JiJoye (EBV2) cell lines (Buisson et al, 1994), was used to select primers and probes for genotyping. BM1 5′-CCACCAAGGCCTACCCGTCCT-3′ and BM2 primers 5′-GTGCTGCTGGTGGTGGCAAT-3′ were used for both subtypes EBV1 and EBV2. The amplified products were 262 bp for EBV1 and 259 bp for EBV2. Amplification of DNA (1 µg) was carried out as previously described (Buisson et al, 1994). The internal oligonucleotides used as probe were 5′-CGCATG-CATCTCCCTGTCTT-3′ for EBV1 and 5′-CCACAAAGGCTCA-CACTAGG-3′ for EBV2.

### In situ hybridization (ISH) procedure

Frozen and paraffin-embedded sections (10 µm) were used for ISH studies. Paraffin-embedded sections were dewaxed in toluene, rehydrated in graded ethanol, digested with 10 µg/ml proteinase K (Roche Molecular Biochemicals, Meylan, France) for 30 min at 37˚C. Sections were mounted on subbed glass slides and prepared for hybridization to RNA probes, as previously described (Ouafik et al, 1990). Radiolabelled riboprobes were prepared using uridine 5′-[α-$^{35}$S-thio] triphosphate (Amersham Pharmacia Biotech, Uppsala, Sweden) and T3 or T7 polymerase (Stratagene, La Jolla, CA) to synthesize, respectively, RNA sense and antisense transcripts of 200 bp EBER1 cDNA contained in pBluscript plasmid (kindly provided by BE Griffin and LG Labrecque). Hybridized sections were exposed to Ilford K5 autoradiography emulsion for a variable length of time (1 week–1 month) to confirm the heterogeneous labelling patterns.

Images were recorded using a PixCell II system (Arcturus Engeneering, Mountain View, USA) which incorporates an Olympus IX-50 microscope and a single sheep CCD colour camera (COHU). Digitalization was performed via the colour frame grabber Matrix Meteor™ slotted to a microcomputer. The public domain software Image, written by W Rosbard at the National Institute of Heath (NIH), was used for the image processing. The mean grain density was quantified by morphological filtering and thresholding, and expressed as percentage of surface for both the tumour epithelial formations and the stroma.

### Laser capture microdissection

OCT embedded tissue blocks prepared from frozen breast cancer samples were sectioned at 8 µm in a cryostat. The sections mounted on uncoated glass slides were fixed in 70% ethanol for 30 s, stained with Mayer's haematoxylin, and washed in 70% and 95% ethanol for 60 s. Subsequently, slides were stained with eosin Y. Staining was followed by two 60 s dehydration steps in 95% and 100% ethanol and two final 15 min dehydration steps in xylene. Once air-dried, the sections were microdissected with a PixCell II LCM system (Arcturus Engeneering, Mountain View, USA). For each tumour analysed, several epithelial areas (approximatively $5 \times 10^3$ cells) were independently captured; stromal areas without infiltrating malignant epithelial cells were pooled to provide a sufficient number of GAPDH copies. Cell populations were estimated to be homogeneous as determined by microscopic

*© 2001 Cancer Research Campaign*

visualization. DNA from laser captured cells was extracted and subsequently used for Q-PCR.

### Statistical analysis

Patients' characteristics were compared with the Chi-square test. $P < 0.05$ was considered significant.

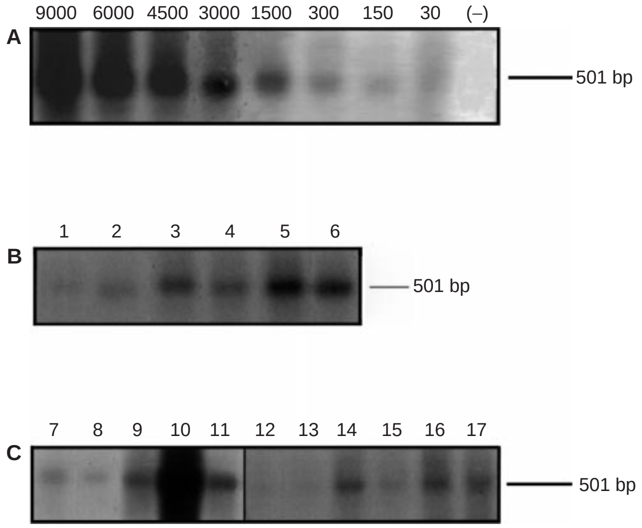

**Figure 1** Presence of EBV in breast cancers. DNA prepared from Namalwa cell line (**A**) (from 30 ng to 0.1 ng) and breast cancer samples (200 ng) from Algeria (**B**), Marseille (**C**; 7–11), Denmark (**C**; 12–17) was subjected to 38 cycles of PCR utilizing primers described under 'Materials and Methods'. Annealing was carried out at 56˚C for 30 s followed by extension at 68˚C for 35 s. The products were fractionated on a 1% agarose gel and prepared for Southern blot analysis using a 200 bp DNA probe as described in 'Materials and Methods'. A control lacking DNA was subjected to PCR at the same time (–). Samples 1, 2, 7, 8, 12, 13, 15: <150; 3, 4, 9, 14, 16, 17: 150–1500; 5, 6, 10, 11: >1500 BamHIC copies/500 ng DNA

### RESULTS

### PCR analysis of EBV genome

In order to detect the presence of EBV genome in breast cancers, genomic DNA was prepared and used for BamHIC PCR analysis. A 501 bp amplified product was obtained (Figure 1). The presence of the BamHIC subregion of the EBV genome was used to define EBV positivity. Among the 509 breast cancers analysed, only 31.8% were EBV positive. The EBV-negative tumours included samples shown with low, moderate and high degree of lympho-plasmocytic infiltration by histopathology. In addition, normal tissues adjacent to breast cancers ($n = 3$) and benign breast tumors ($n = 7$), were investigated by PCR and were all found to be negative.

Breast cancers showed a positive ratio from Algeria of 40.0%, and from Tunisia 32.8%, Marseille 26.7%, St Cloud 29.2%, The Netherlands 32.5% and Denmark 35.1%, respectively. Frequencies were not different among the geographical areas studied. However, EBV-positive breast cancers from the NPC high- and intermediate-risk areas showed higher loads of the EBV genome than those from the low-risk areas: that is 47.0% and 28.1% of the samples contained more than 150 BamHIC copies/500 ng DNA, respectively ($P = 0.01$) and 22.7% and 8.3% more than 1500 BamHIC copies/500 ng DNA ($P = 0.01$) (Table 2).

### Detection of EBV genome and tumour characteristics

In the overall population, no significant link was observed between the detection of the EBV genome by BamHIC PCR and age, menopausal status, tumour size and lymph node involvement. No association was also demonstrated with histoprognostic grade; in Marseille the highest EBV frequency was found in grade 3 (35.6%) and the lowest in grade 1 (22.2%), but differences were not significant. The load of the EBV genome was also not correlated with clinical and pathological characteristics. It is important

**Table 2** Frequency and load of EBV genome, as determined by qualitative and semi-quantitative BamHIC PCR respectively, in the overall population of breast cancers and in breast cancers from different geographical areas

| | EBV + tumours | | EBV + tumours with load of EBV genome >150[4] | | | | EBV + tumours with load of EBV genome >1500[4] | | | |
|---|---|---|---|---|---|---|---|---|---|---|
| Population | % vs all tumours | | % vs all tumours | | % vs EBV + tumours | | % vs all tumours | | % vs EBV + tumours | |
| Total | 31.8 | (162/509) | 11.4 | (58/509) | 35.8 | (58/162) | 4.5 | (23/509) | 14.2 | (23/162) |
| Algeria | 40.0 | (16/40) | 17.5 | (7/40) | 43.8 | (7/16) | 10.0 | (4/40) | 25.0 | (4/16) |
| Tunisia | 32.8 | (19/58) | 13.8 | (8/58) | 42.1 | (8/19) | 6.9 | (4/58) | 21.1 | (4/19) |
| Marseille | 26.7 | (31/116) | 13.8 | (16/116) | 51.6 | (16/31) | 6.0 | (7/116) | 22.6 | (7/31) |
| St Cloud | 29.2 | (21/72) | 11.1 | (8/72) | 38.1 | (8/21) | 4.2 | (3/72) | 14.3 | (3/21) |
| The Netherlands | 32.5 | (41/126) | 11.9 | (15/126) | 36.6 | (15/41) | 4.0 | (5/126) | 12.2 | (5/41) |
| Denmark | 35.1 | (34/97) | 4.1 | (4/97) | 11.8 | (4/34) | 0.0 | (0/97) | 0.0 | (0/34) |
| NPC high-risk areas[1] | 35.7 | (35/98) | 15.3 | (15/98) | 42.9 | (15/35) | 8.2 | (8/98) | 22.9 | (8/35) |
| NPC intermediate-risk area[2] | 26.7 | (31/116) | 13.8 | (16/116) | 51.6 | (16/31) | 6.0 | (7/116) | 22.6 | (7/31) |
| NPC high- and intermediate-risk areas | 30.8 | (66/214) | 14.5 | (31/214) | 47.0[5] | (31/66) | 7.0 | (15/214) | 22.7[6] | (15/66) |
| NPC low-risk areas[3] | 32.5 | (96/295) | 9.2 | (27/295) | 28.1 | (27/96) | 2.7 | (8/295) | 8.3 | (8/96) |

[1]Algeria and Tunisia. [2]Marseille. [3]St Cloud, The Netherlands and Denmark. [4]Expressed as the number of BamHIC copies/500 ng DNA. [5,6]Significant difference was observed by Chi-square test between NPC high-, intermediate-risk areas, and low-risk areas ($P = 0.01$). [7]EBV+ tumours, tumours positive for EBV: NPC, nasopharyngeal carcinoma.

 *© 2001 Cancer Research Campaign*

to point out that 40.0% of each inflammatory and non-inflammatory Algerian samples were EBV positive; only inflammatory carcinomas (41.7% vs 28.6%) showed high rate of EBV positivity in Tunisia.

### EBV genotyping

EBV genotyping was applied to 13 breast cancers from Algeria, Marseille, the Netherlands and Denmark containing more than 150 BamHIC copies/500 ng DNA. EBV1 was present in all the tumours examined. EBV2 was detected in 2 EBV1-positive samples from the Netherlands (not shown).

### In situ hybridization

20 EBV-positive breast cancers from Algeria, Tunisia and Marseille were prepared for ISH studies in order to determine whether EBV expression occurs in the tumour epithelial compartment or in lymphoplasmocytic cells infiltrating the stroma. Positive results were obtained on the malignant epithelial compartment of those tumours with more than 1500 BamHIC copies/500 ng DNA ($n = 10$). Considerable heterogeneity was however observed among malignant epithelial areas. Heterogeneity was already detectable using 1 week exposure and was still evident when the exposure time was extended to 1 month. Figure 2A shows a section hybridized with an antisense probe. Silver grains were concentrated over the tumour epithelial formations, whereas the stroma displayed fewer isolated grains; mean grain density was 9.4% and 1.7%, respectively. Such a pattern was considered to express the specific localization of EBV material, since the close adjacent section hybridized with the sense riboprobe, representative of the technical background for the exposure time, also contained a low density of randomly distributed silver grains both over the tumour epithelial formations (1.9%) and the stroma (1.2%) (Figure 2B). Figures 2C and 2D show two distant fields of the same highly heterogeneous section hybridized with the antisense riboprobe. The epithelial areas in field 2C contained a high density of silver grains (9.5%) corresponding to EBV positivity whereas the low density (1.8%) obtained in field 2D corresponds to negativity. For both fields 2C (1.5%) and 2D (0.9%), a low grain density was observed in the stroma. For the same tumours, signal intensity was lower in paraffin-embedded sections than in cryosections.

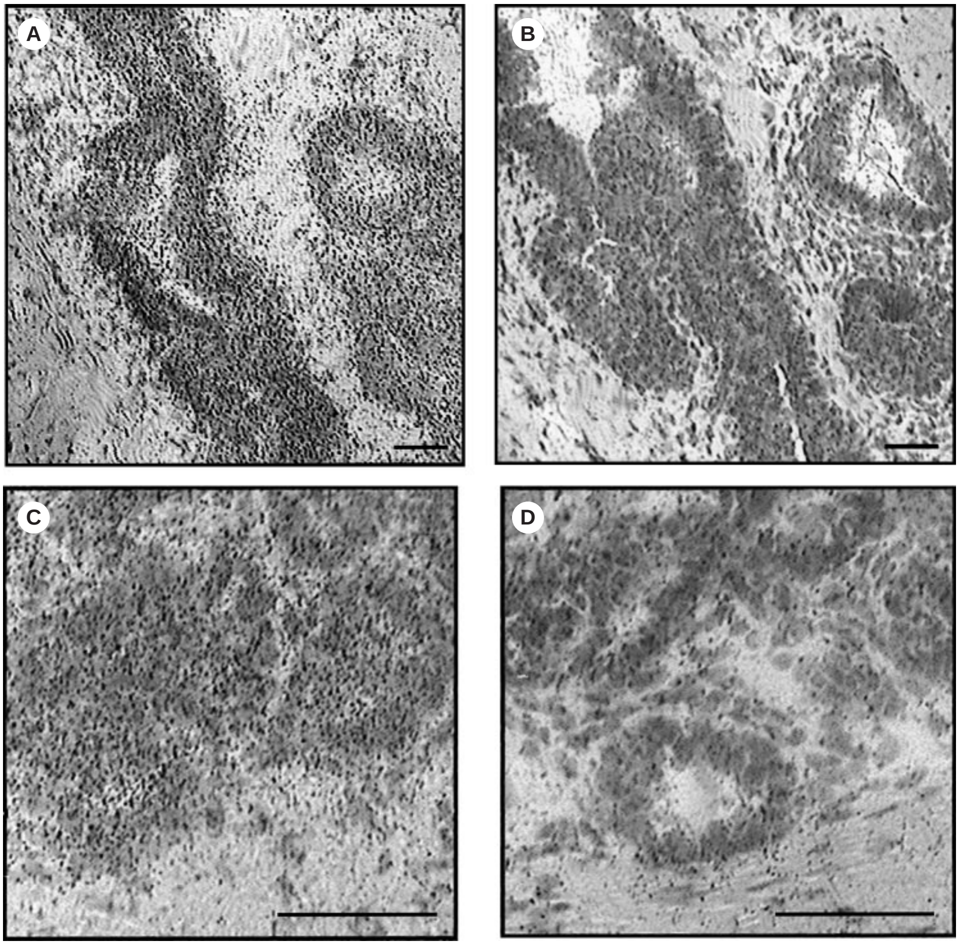

**Figure 2** In situ hybridization for EBV in breast cancer. Cryostat tumour sections (10 μm-thick) were hybridized with $^{35}$S-labelled antisense and sense riboprobes, dipped in photographic emulsion, exposed for 1 month, developed, and lightly counterstained with haematoxylin. (**A**) shows a section hybridized with the antisense riboprobe. Silver grains are concentrated over the tumour epithelial formations. A close adjacent section from the same tumour shown in (**B**), was hybridized with the sense riboprobe and displays a low density of randomly distributed silver grains. (**C**) and (**D**) show two distant fields of the same section hybridized with the antisense riboprobe. The epithelial areas in field **C** contain a much higher density of silver grains compared to those in field (**D**). Calibration bar for all micrographs = 50 μm

© 2001 Cancer Research Campaign

8 of the breast cancers, shown by PCR to be EBV negative were examined by ISH and showed non-specific labelling.

### Laser microdissection combined with real-time quantitative PCR

In order to perform precise molecular analysis of purified cell populations and determine whether EBV is localized in the stroma or in the tumour epithelial compartment, as suggested by ISH, two EBV-positive breast cancers from Marseille were analysed combining LCM and Q-PCR. Figure 3 shows a target tissue before laser shots (Figure 3A) and the captured malignant epithelial cells (Figure 3B). In both tumours some malignant epithelial areas were highly positive, with EBV loads ranging from 128 to 5816 BamHIC copies/100 ng GAPDH; however, other malignant epithelial areas independently procured from the same case were EBV-negative. GAPDH did not significantly differ between positive and negative epithelial areas. The stroma areas were EBV-negative (Table 3).

**Table 3** Load of EBV genome, as determined by real-time quantitative BamHIC PCR in epithelial and stromal cell populations captured by laser microdissection from two EBV-positive breast cancers

| Sample | Cell population | GAPDH[1] | EBV genome[2] |
|---|---|---|---|
| A | Epithelium | 11.3 | 5816 |
| A | Epithelium | 6.6 | 1956 |
| A | Epithelium | 35.4 | 128 |
| A | Epithelium | 7.1 | 0 |
| A | Epithelium | 19.3 | 0 |
| A | Epithelium | 33.1 | 0 |
| A | Stroma | 6.6 | 0 |
| B | Epithelium | 56.8 | 1532 |
| B | Epithelium | 19.3 | 403 |
| B | Epithelium | 29.0 | 272 |
| B | Epithelium | 6.6 | 0 |
| B | Epithelium | 14.8 | 0 |
| B | Stroma | 2.6 | 0 |

[1]Expressed as the number of GAPDH copies. [2]Expressed as the number of BamHIC copies/100 ng GAPDH.

### DISCUSSION

Our results demonstrate that the EBV genome can be detected in some breast cancers with a predominance of the EBV1 subtype. Overall, 31.8% of our 509 tumour samples were positive for the BamHIC non repetitive sequence. Labrecque et al (1995) reported conservatively that 21% of breast cancers were positive for the BamHIW repeat sequence, compared to 51% in Bonnet's study (1999). Elsewhere, absence of BamHIW has been reported in 10 medullary carcinomas (Lespagnard et al, 1995). In 37 tumours analysed in the current study (not shown), the concordance between BamHIC and BamHIW primer pairs for PCR analyses was 83.7%.

In our overall population, presence of the EBV genome was not correlated with age, menopausal status, tumour size, nodal status, or histological grade. In Marseille, there was however a trend toward a positive association between EBV and grade, as reported by Bonnet et al (1999). High rates of positive tumours were observed in young Algerian patients and in inflammatory samples.

The ISH using a $^{35}$S-labelled riboprobe for EBERs demonstrated that EBV expression was exclusively localized in malignant epithelial cells. The samples found strongly positive by PCR were also positive by ISH. Within individual tumours, a high heterogeneity was however observed among epithelial cell clusters. No labelling was obtained for samples that were negative by PCR. In our study radioactive ISH signal intensities were low in paraffin-embedded sections. The absence of labelling that has been reported by others (Chang et al, 1992; Lespagnard et al, 1995; Glaser et al 1998) could be a result of technical problems, such as the fixation as reported by Penault-Llorca et al (1994), or of the low sensitivity of non-radioactive ISH.

Laser capture microdissection (LCM) was combined with Q-PCR to quantify the EBV genome in specific cell populations of the tumours. This is the first time that this technique has been used to localize EBV in breast cancers. The use of LCM clearly confirmed the epithelial localization of EBV and the heterogeneity among epithelial cells, as suggested by ISH. The highest loads of EBV genome found here in malignant epithelial populations captured from EBV-positive breast cancers are in the median range of those observed for NPC collected in Maghrebian countries, when both are expressed as the number of BamHIC copies/100 ng GAPDH (not shown).

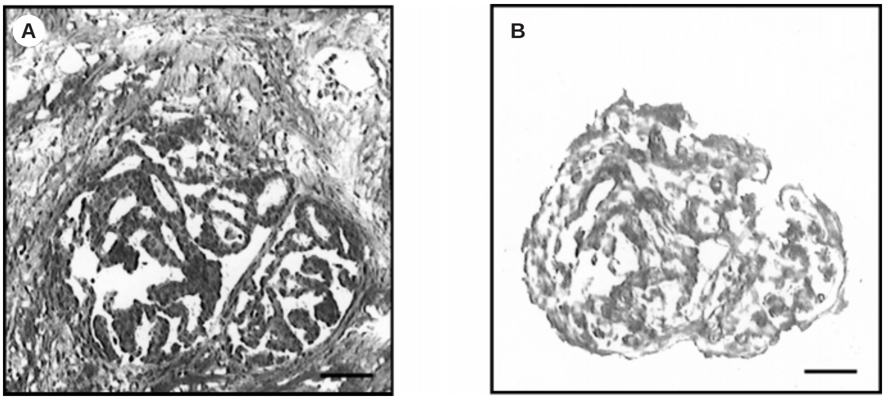

**Figure 3** Laser capture microdissection (LCM) in breast cancer. Cryostat tumour sections (8 μm thick) stained with Mayer's haematoxylin and eosin Y were microdissected with the Arcturus Pixcell II system to procure homogeneous cell populations. **A** shows a section before LCM. **B** shows the malignant epithelial cells captured by LCM. Calibration bar for all micrographs = 50 μm

*© 2001 Cancer Research Campaign*

It is unlikely that our PCR-positive results are due to contamination. First, EBV was detected in 2 independent amplifications, semi-quantitative PCR with Southern blot hybridization and Q-PCR combined with LCM; secondly, Q-PCR was run in a closed-tube system and required no post-amplification manipulation; finally, normal human genomic DNA (Roche Molecular Biochemicals, Meylan, France) and controls lacking DNA always remained negative in PCR analyses.

Detection of EBV in breast cancer has engendered a great deal of controversy. In a recent note, Brink et al (2000) suggested that positivity with EBV is most likely caused by the presence of some infected lymphocytes in the tumour samples. However, it is possible that the RT-PCR used by these authors was not sensitive enough to detect all the EBV transcripts (Joab, 2000). In our study, the presence of EBV in tumoral lymphoplasmocytic infiltration can be ruled out. First, ISH demonstrated no specific labelling of the stroma and the infiltrating lymphoplasmocytic cells near the positive tumoral epithelial clusters; secondly, LCM combined with Q-PCR also showed that EBV localization was restricted to some tumour epithelial cell clusters; thirdly, the tumours found to be EBV negative using BamHIC PCR included samples with low, moderate, and high degrees of lymphoplasmocytic infiltration; finally, EBV was not detected in normal tissues adjacent to breast cancers and benign breast tumours, in agreement with previous studies (Labrecque et al, 1995; Bonnet et al, 1999).

One hypothesis mentioned by Labrecque et al (1995) for the inconsistency concerning the presence of EBV in breast cancer is that EBV-positive tumours might belong to a subgroup of breast cancers. Our study shows that EBV frequencies were not different among the geographical areas analysed. In contrast, the load of EBV genome was significantly higher in NPC high-risk areas (Maghrebian countries), and intermediate-risk areas (southern France) where migrants of Maghrebian origin and French people born in Maghreb account for a large proportion of the population. Although differences in the patient characteristics among the participating centres suggest that caution should be used in making comparisons by country, environmental factors inherent to life style and food habits that are not modified by migration, and inherit susceptibility (Steinitz et al, 1990; Bouchardy et al, 1996; Parkin and Iscovich, 1997), may partly explain the observed differences.

In summary, we confirm the presence of EBV in breast cancers. Our findings demonstrate that at least 31.8% of 509 samples of invasive ductal carcinomas contain a subregion of BamHIC, encoding EBERs, and that the load of intratumoral EBV genome differs according to the geographical area. ISH and LCM studies show that the EBV is located in some tumour epithelial cell clusters, with no specific detection in lymphoplasmocytic infiltrations.

### ACKNOWLEDGEMENTS

This work was supported by a grant from *Assistance Publique de Marseille* (AORC 1998; UF 2843). We wish to thank Drs BE Griffin and LG Labrecque for providing the EBER1 DNA. Miss A Durand, Miss H Gérard, Mr O Guirou and Mr M Soullière are acknowledged for their skillful technical assistance. We also thank Dr A Lachard for performing LCM.

### REFERENCES

Andersen KW, Mouridsen HT, Castberg T, Fischerman K, Andersen J, Hou-Jensen K, Brincker H, Johansen H, Henriksen E, Rorth M and Rossing N (1981) Organisation of the Danish adjuvant trials in breast cancer. *Dan Med Bull* **28**: 102–106

Bonnet M, Guinebretiere JM, Grunewald V, Kremmer E, Grunewald V, Benhamou E, Contesso G and Joab I (1999) Detection of Epstein–Barr virus in invasive breast cancers. *J Natl Cancer Inst* **91**: 1376–1381

Bouchardy C, Parkin DM, Wanner P and Khlat M (1996) Cancer mortality among north African migrants in France. *Int J Epidemiol* **25**: 5–13

Brink AA, Van den Brule AJ, Van Diest P and Meijer CJ (2000) Re: detection of Epstein-Barr virus in invasive breast cancers. *J Natl Cancer Inst* **92**: 655–656

Buisson M, Morand P, Genoulaz O, Bourgeat MJ, Micoud M and Seigneurin JM (1994) Changes in the dominant Epstein–Barr virus type during human immunodeficiency virus infection. *J Gen Virol* **75**: 431–437

Bustin SA (2000) Absolute quantification of mRNA using real-time reverse transcription polymerase chain reaction assays. *J Mol Endocrinol* **25**: 169–193

Chang KL, Chen YY, Shibata D and Weiss LM (1992) Description of an in situ hybridization methodology for detection of Epstein-Barr virus RNA in paraffin-embedded tissues, with a survey of normal and neoplastic tissues. *Diagn Mol Pathol* **1**: 246–255

De Thé G (1993) Epidemiology, pathogenesis and prevention of EBV associated malignancies. In The Epstein–Barr virus and associated diseases, Tursz T, Pagano JS, Ablashi DV, de Thé G, Lenoir G and Pearson GR (eds.), pp 15–30. Colloque INSERM, Vol 225, John Libbey Eurotext Ltd

Gaffey MJ, Frierson HFJr, Mills SE, Boyd JC, Zarbo RJ, Simpson JF, Gross JK and Weiss LM (1993) Medullary carcinoma of the breast. Identification of lymphocyte subpopulations and their significance. *Mod Pathol* **6**: 721–728

Glaser SL, Ambinder RF, DiGiuseppe JA, Horn-Ross PL and Hsu JL (1998) Absence of Epstein-Barr virus EBER-1 transcripts in an epidemiologically diverse group of breast cancers. *Int J Cancer* **75**: 555–558

Horiuchi K, Mishima K, Ohsawa M and Aozasa K (1994) Carcinoma of stomach and breast with lymphoid stroma: localisation of Epstein-Barr virus. *J Clin Pathol* **47**: 538–540

Hubert A, Jeannel D, Tuppin P and de Thé G (1993) Anthropology and epidemiology: a pluridisciplinary approach of environmental factors of nasopharyngeal carcinoma. In: Tursz T, Pagano JS, Ablashi DV, de Thé G, Lenoir G and Pearson GR (eds.), *The Epstein–Barr virus and associated diseases*, pp 775–788. Colloque INSERM, Vol 225, John Libbey Eurotext Ltd

Epstein-Virus and Kaposi's sarcoma herpesvirus/human herpes virus (1997) 8. IARC monographs on the evaluation of carcinogenic risks to humans, volume 70: Lyon

Jeannel D, Ghnassia M, Hubert A, Sancho-Garnier H, Eschwege F, Crognier E and de-The G (1993) Increased risk of nasopharyngeal carcinoma among males of French origin born in Maghreb (north Africa). *Int J Cancer* **54**: 536–539

Joab I (2000) Response: re: detection of Epstein-Barr virus in invasive breast cancers. *J Natl Cancer Inst* **92**: 656

Labrecque LG, Barnes DM, Fentiman IS and Griffin BE (1995) Epstein–Barr virus in epithelial cell tumors: a breast cancer study. *Cancer Res* **55**: 39–45

Lespagnard L, Cochaux P, Larsimont D, Degeyter M, Velu T and Heimann R (1995) Absence of Epstein–Barr virus in medullary carcinoma of the breast as demonstrated by immunophenotyping, in situ hybridization and polymerase chain reaction. *Am J Clin Pathol* **103**: 449–452

Ouafik L, May V, Keutmann HT and Eipper BA (1989) Developmental regulation of peptidylglycine alpha-amidating monooxygenase (PAM) in rat heart atrium and ventricle. Tissue- specific changes in distribution of PAM activity, mRNA levels, and protein forms. *J Biol Chem* **264**: 5839–5845

Ouafik L, May V, Saffen DW and Eipper BA (1990) Thyroid hormone regulation of peptidylglycine alpha-amidating monooxygenase expression in anterior pituitary gland. *Mol Endocrinol* **4**: 1497–1505

Parkin DM (1986) *Cancer occurrence in developing countries*. Vol 75, IARC: Lyon

Parkin DM and Iscovich J (1997) Risk of cancer in migrants and their descendants in Israel: II. Carcinomas and germ-cell tumours. *Int J Cancer* **70**: 654–660

Penault-Llorca F, Adelaide J, Houvenaeghel G, Hassoun J, Birnbaum D and Jacquemier J (1994) Optimization of immunohistochemical detection of ERBB2 in human breast cancer: impact of fixation. *J Pathol* **173**: 65–75

Rickinson AB, Young LS and Rowe M (1987) Influence of the Epstein–Barr virus nuclear antigen EBNA 2 on the growth phenotype of virus-transformed B cells. *J Virol* **61**: 1310–1317

Romain S, Martin PM, Klijn JG, van Putten WL, Look MP, Guirou O and Foekens JA (1997) DNA-synthesis enzyme activity: a biological tool useful for predicting anti-metabolic drug sensitivity in breast cancer? *Int J Cancer* **74**: 156–161

Sambrook J, Fritsch EF and Maniatis T (1989) *Molecular cloning: a laboratory manual*. Cold Spring Harbor: Cold Spring Harbor Laboratory

Sobin LH and Wittekind Ch (1997) *UICC International Union Against Cancer. TNM classification of malignant tumors*. Wiley-Liss: New York

© 2001 Cancer Research Campaign 

Steinitz R, Iscovich JM and Katz L (1990) Cancer Incidence in young offspring of Jewish immigrants to Israel. A methodological study. I. Nasopharyngeal malignancies and Ewing sarcoma. *Cancer Detect Prev* **14**: 547–553

Tabbane F, Muenz L, Jaziri M, Cammoun M, Belhassen S and Mourali N (1977) Clinical and prognostic features of a rapidly progressing breast cancer in Tunisia. *Cancer* **40**: 376–382

Tabbane F, el May A, Hachiche M, Bahi J, Jaziri M, Cammoun M and Mourali N (1985) Breast cancer in women under 30 years of age. *Breast Cancer Res Treat* **6**: 137–144

zur Hausen H (1991) Viruses in human cancers. *Science* **254**: 1167–1173

 *© 2001 Cancer Research Campaign*
